# Acaricidal Efficacy of Biosynthesized Zinc Oxide Nanoparticles Against *Hyalomma dromedarii* (Acari: Ixodidae) and Their Toxic Effects on Swiss Albino Mice

**DOI:** 10.1007/s11686-022-00530-8

**Published:** 2022-03-22

**Authors:** Hoda S. M. Abdel-Ghany, Sobhy Abdel-Shafy, Mai M. Abuowarda, Rabab M. El-Khateeb, Essam M. Hoballah, Magdy M. Fahmy

**Affiliations:** 1 Department of Parasitology and Animal Diseases, Veterinary Research Institute, National Research Centre, Dokki, Giza, Egypt; 2 Department of Parasitology, Faculty of Veterinary Medicine, Cairo University, Giza, Egypt; 3 Department of Agriculture Microbiology, Agricultural, and Biological Research Institute, National Research Centre, Dokki, Giza, Egypt

**Keywords:** Camel, *Hyalomma dromedarii*, *Melia azedarach*, Zinc oxide nanoparticles, SEM–EDS, Toxicity

## Abstract

**Purpose:**

The current study aimed to investigate the efficacy of zinc oxide nanoparticles (ZnO NPs) synthesized by *Melia azedarach* aqueous extract to control *Hyalomma dromedarii* tick, and to evaluate their toxic effects on Swiss albino mice.

**Methods:**

ZnO NPs were synthesized using *M. azedarach* aqueous extract. UV–visible spectroscopy, Fourier transform infrared spectroscopy, scanning electron microscopy, and energy-dispersive spectroscopy were used to characterize the synthesized NPs. Egg, nymph, larva, and adult immersion tests were used for bioassay of tick stages with the synthesized ZnO NP. A toxicity study was performed on Swiss albino mice after treatment with 1/10 of the oral LD_50_ of ZnO NPs (8437 mg/kg) for 5 successive days by oral gavage.

**Results:**

The LC_50_ of ZnO NPs on the eggs, larvae, and nymphs was 11.6, 8.03, and 3.9 mg/ml, respectively. The reproductive performance of females treated with ZnO NPs was lower than that of untreated females. The hematological results showed an insignificant increase in the level of white blood cells with normal red blood cells, hemoglobin, in addition to normal platelet count. The biochemical analysis showed an insignificant increased level (*P* > 0.05) of alkaline phosphatase and alanine aminotransferase. The liver and kidney suffered few histopathological changes after oral administration of ZnO NPs.

**Conclusion:**

These results suggest that ZnO NPs have good acaricidal activity against eggs, larvae, and engorged nymphs of *H. dromedarii.* ZnO NPs minimized the number of eggs laid by engorged females and the hatchability of their eggs. ZnO NPs did not affect unfed adults. The toxicity results of the mice revealed insignificant changes in the hemogram, biochemistry, with liver and kidney suffering few histopathological changes. Future studies are needed to assess application routes (topical vs oral). Based on these findings, ZnO NPs may be incorporated in the control of camel tick *H. dromedarii.*

## Introduction

Nanotechnology is one of the fastest-growing technologies to produce biomaterials with controlled shape and size. Due to their small size, nanoparticles (NPs) are extensively used in many fields (*e.g*., electronics, catalysis, gas sensing, and environmental remediation) and incorporated into various commercial products, biotechnology, and biomedical applications [1, 2]. Progress in the use of metal oxide NPs in environmental applications has encouraged their synthesis. Zinc oxide nanoparticles (ZnO NPs) are the most promising inorganic metal oxide that is extensively used due to their suitable magnetic, electrical, and optical properties [3].

Several approaches are available for synthesizing ZnO NPs, including hydrothermal synthesis, vapor transport, precipitation, and sol–gel synthesis. However, these approaches have certain disadvantages as the use of toxic chemicals, expensive equipment, and application of high temperature, energy, and pressure are required for synthesization of NPs [4, 5]. The biosynthesis of metal oxide NPs by plant-derived materials has been preferred over physical and chemical methods, and even those using microorganisms, due to the high rate of synthesis and because the synthesis environment does not need to be aseptic [6]. In addition to these benefits, green synthesis has also been proven to be extremely useful in obtaining NPs with controlled size and shape [7]. Several studies reported that the plant extracts have secondary metabolites as enzymes, vitamins, amino acids, flavonoids, and phenolic compounds which allow reduction, capping, and stabilization of synthesized NPs [8].

*Melia azedarach* (family: Meliaceae) is a native plant for most tropical–subtropical regions, many African and Arab countries [9]. Aqueous extracts of *M. azedarach* leaves have been successfully used in the biosynthesis of different NPs as ZnO NPs [10], silver NPs [11], and CuO NPs [12].

Ticks are one of the most destructive blood-sucking ectoparasites of livestock and wild animals. Heavy infestations of livestock by ticks result in huge economic losses through severe blood loss, anorexia, stress, irritation, toxicosis, and the transmission of pathogens, which lead to decreases in productivity, along with the cost of treatments and animal death [13, 14]. Ticks and tick-borne diseases cause worldwide annual losses of about $7 billion [15].

*Hyalomma dromedarii* is the predominant ixodid tick infesting camels. It is considered one of the crucial problems for camel production in numerous regions of the Middle East [16]. The worldwide control of tick is mainly based on the repeated use of acaricides, which has led to environmental pollution, and resistance development, increasing the cost of control [17]. Therefore, new strategies concerning tick control are needed to limit the drawbacks of chemical acaricides. Nano-biotechnology is considered a novel approach in the treatment of arthropod vectors and parasitic infections [18]. Synthesized ZnO NPs have been proven to have acaricidal activity against *Rhipicephalus (Boophilus) microplus* [19–21], in addition to pediculocidal activity against *Pediculus humanus capitis* (Phthiraptera: Pediculidae) and larvicidal activity against mosquitoes [19].

*In vivo* experiments are required to demonstrate NPs toxicity in the biological systems. Nanoparticles can enter the body through ingestion, inhalation, and injection [22]. They may be transmitted to the blood causing adverse effects in different organs [23]. Experiments of NPs biodistribution showed that the liver, kidney, and spleen were the main sites after oral administration [24].

This study aimed to (1) assess the *in vitro* efficacy of green-synthesized ZnO NPs against all *H. dromedarii* stages, and (2) evaluate the toxic effects of oral administration of ZnO NPs in mice.

## Materials and Methods

### Preparation of Aqueous *Melia azedarach* Extract

*Melia azedarach* dried ripened fruits were obtained from the Genetics and Cytology Department, Biotechnology Research Institute, National Research Centre, and identified according to El-Hadidi and Boulos [25]. The fruits were pulverized to a fine powder using a stainless-steel knife mill. To prepare an aqueous extract of *M. azedarach* fruits, about 15 g of the fruits was mixed in 100 ml of double-distilled water, which was then heated at 70 °C for 30 min, cooled, filtered (first by muslin cloth followed by filtration through Whatman filter paper number 1), then stored at 4 °C.

### Green Synthesis of Zinc Oxide Nanoparticles

For synthesizing ZnO NPs, 0.1 M zinc nitrate hexahydrate [Zn (NO_3_)_2_6 H_2_O] was freshly prepared using distilled water. After that, *M. azedarach* aqueous extract (20 ml) was mixed with prepared zinc nitrate (80 ml). The pH was adjusted up to 7 using 2 N NaOH then subjected to continuous stirring (60 °C, 1,400 rpm, 6 h). After the incubation period, the appearance of yellow color was the indication for ZnO NPs synthesis. The solution was subjected to centrifugation (5000 rpm, 20 min), the supernatant was discarded and distilled water was added to the pellets for another centrifugation. This process was repeated twice to remove the impurities. The obtained pellets were oven-dried (80 °C, 8 h), then calcined in Muffle Furnace (400 °C, 2 h). After drying, the samples were kept in air-tight bottles until characterization [26].

### Characterization of the Green-Synthesized Zinc Oxide Nanoparticles

For characterization of ZnO NPs, UV–Vis spectroscopy, Fourier transform infrared spectroscopy, scanning electron microscopy, and energy-dispersive spectroscopy were used.

UV–Vis analysis was conducted by UV–Vis spectroscopy (JASCO V-730 UV–Vis spectrophotometer, NRC) through a scanning range of between 200 and 600 nm wave length. FTIR spectrometer (JASCO 6100-FTIR, NRC) was used to determine the FTIR spectra of the synthesized ZnO NPs within the scan range of 400–4000 cm^−1^ resolution. In addition, FTIR identified the functional groups of *M. azedarach* aqueous extract that responsible for the formation of nanoparticles. SEM and EDS were used to identify the morphology, size, and elemental composition of synthesized ZnO NPs. The sample was placed on a carbon-coated copper grid and the images were analyzed by SEM and EDS (QUANTA FEG-250, NRC).

### Ticks

Engorged females of *H. dromedarii* were collected in wide-mouth glass jars sealed with muslin cloth from naturally infested *Camelus dromedarius* at Berkash market (30°09′58.4″ N, 31°02′13.2″ E) Giza, Egypt. After that, the obtained samples were identified following the key of Walker *et al.* [27]. Each female was kept in a separate plastic cup and maintained at 25 ± 1 °C and 75–80% RH until oviposition. Daily collection of eggs was performed, which were kept in the incubator (Friocell, MMM, Germany). Embryonated eggs, larvae, engorged nymphs, unfed adults, and engorged females which were used in the bioassay were obtained according to the method described by Abdel-Ghany *et al.* [28].

### Effect of Synthesized Zinc Oxide Nanoparticles

At first, the synthesized ZnO NPs were diluted to the desired concentrations using 2% Tween 80. Tween 80 (2%) was used as solvent control while (Deltamethrin) 1 ml/l was used as reference acaricide. Butox^®^ 5.0 was selected because it is commercially used in the field in Egypt. All treatment solutions were sonicated for 5 min before treatment to avoid settling of the synthesized nanoparticles especially at higher concentrations. The dipping method (immersion test) was used in the bioassay experiments because in the large herds the application method is the dipping of animals in the recommended synthetic acaricide for one minute. Therefore, this method was used in both Butox^®^ 5.0 and ZnO NPs to mimic the field application. The pilot test was conducted to detect the suitable concentrations of ZnO NPs for each tick stage in the bioassay experiments. The pilot test depends on testing folded concentrations starting from the highest one which gave 100% mortality to the lowest one which gave zero mortality. Following this, the concentrations that gave lower and higher than 50% mortalities were chosen for the bioassay experiments.

### Egg Immersion Test (EIT)

Approximately, 300 embryonated eggs of *H. dromedarii* were immersed for 1 min in 1 ml of each concentration (4, 8, 16, and 32 mg/ml) of the synthesized ZnO NPs following the method described by Abdel-Ghany *et al*. [29]. Each concentration was replicated 3 times (100 eggs/replicate). Eggs were taken from the same female obtained from the established colony. After that, the treated eggs were incubated for 14 days to determine the effect of ZnO NPs on these eggs. The mortality percentage of eggs was calculated by counting dead eggs and hatched larvae.

### Larval Immersion Test (LIT)

In LIT, 2-week-old *H. dromedarii* larvae were immersed at different concentrations of synthesized ZnO NPs. Approximately, 300 larvae (for each concentration) were immersed for 1 min in 1 ml of the tested concentrations (4, 8, 16, and 32 mg/ml) of the synthesized ZnO NPs according to the method described by Abdel-Ghany *et al*. [30]. Each concentration was replicated 3 times (100 larvae/replicate). After treatment, the larvae were incubated at 25 ± 1 °C, relative humidity of 75–80%. After 24 h, the dead larvae were counted for calculation of the mortality rate.

### Nymphal Immersion Test (NIT)

Thirty engorged nymphs for each concentration were used in the NIT to test the efficacy of ZnO NPs according to the method of Abdel-Ghany *et al.* [29]. The engorged nymphs were used instead of the unfed nymph as *H. dromedarii* on rabbits behaved as two host, so we cannot obtain unfed nymphs, and consequently engorged nymphs were used in the bioassay. The used concentrations were (2, 4, 8, and 16 mg/ml), 5 ml of each concentration was used for the immersion for 1 min. Each concentration was replicated 3 times. After that, tested nymphs were incubated at 25 ± 1 °C, relative humidity of 75–80% until molting occurred around 20 days. The mortality percentage of nymphs that failed to molt was calculated.

### Adult Immersion Test (AIT)

#### Unfed Adults

In a pilot test, folded concentrations from the lowest 4 mg/ml to the highest 32 mg/ml concentration gave no mortality. Therefore, in the bioassay experiment, unfed adults (10-day old) of *H. dromedarii* were exposed only to the highest concentration of synthesized ZnO NPs (32 mg/ml). Thirty unfed adults (equal number of males and females) were immersed for 1 min in 5 ml of the tested concentration (32 mg/ml). This concentration was replicated three times (10 unfed adults/replicate). After that, the tested adults were kept in an incubator 25 ± 1 °C and relative humidity of 75–80%. The mortality was recorded daily for 7 days.

#### Engorged Females

Although the pilot test gave no mortality in engorged females exposed to ZnO NPs, the bioassay experiment on the engorged females was conducted to follow their reproductive performance representable by egg mass, egg number, and hatchability. To evaluate the efficacy of ZnO NPs on the reproductive performance of engorged females obtained from feeding on rabbits, AIT was used following the method of Drummond *et al*. [31] with some modification. Before treatment, the initial weight of each female was recorded. Nine engorged females were immersed in 10 ml of each tested concentration (4, 8, 16, and 32 mg/ml) of the synthesized ZnO NPs. Each concentration was repeated three times (3 engorged females/replicate). Tested females were incubated at 25 ± 1 °C and 75–80% RH to follow their egg laying. The laid eggs were followed for 15 days to evaluate the Egg Productive Index (EPI) and the hatchability of laid eggs which were calculated as follows:

$${\text{Egg Productive Index (EPI)}} = \,{\text{weight of eggs laid (g)}}/{\text{weight of females }}({\text{g}}).$$

Percentage of hatchability = Number of hatched larvae/number of laid eggs × 100 (Abuowarda *et al*. [32]).

### Toxicity Evaluation of ZnO NPs on Swiss Albino Mice

Thirty adult male mice weighing 20–25 g were obtained and housed in the animal house, National Research Centre in a ventilated room (26 ± 2 °C, 44–56% RH) and provided with good food and water. The experimental design included two groups: the control group and the treatment group. The control group was subdivided into 2 subgroups: the control group (10 mice) left without treatment and the solvent control group (10 mice) administered 2% Tween 80.

The treatment group (10 mice) was administered (844 mg/kg) 1/10 of the oral LD_50_ of ZnO NPs (8,437 mg/kg) by oral gavage according to Mesallam *et al*. [33] for 5 consecutive days. After oral administration of ZnO NPs, any behavioral changes in their activity, food and water uptake, or appearance of toxicity signs on the mice were recorded every day.

Five days after the last administration, blood and sera were collected from mice. The blood samples were used to determine the hemogram and the sera were used to estimate alanine aminotransferase (ALT), alkaline phosphatase (ALP), and creatinine following the methods described by Abdel-Ghany *et al.* [34]. In addition, sections from the liver and kidney of three mice were prepared for histopathological examination as the method described by Abdel-Ghany *et al.* [34].

### Statistical Analyses

Mortality percentages of eggs, larvae, and engorged nymphs*;* reproductive performance of engorged females (including EPI, egg number, and hatchability %) exposed to ZnO NPs, as well as, hematological and biochemical parameters of the mice administered ZnO NPs were statistically analyzed by one-way ANOVA test using F-test followed by Duncan test using SPSS program version 20. The lethal concentration LC_50_ values of eggs, larvae, and engorged nymphs were determined by applying regression equation analysis to the probit transformed data of mortality. The dose–response data were analyzed by probit method [35] using Ehab software.

## Results

### Characterization of Synthesized ZnO NPs

**Fig. 1:**
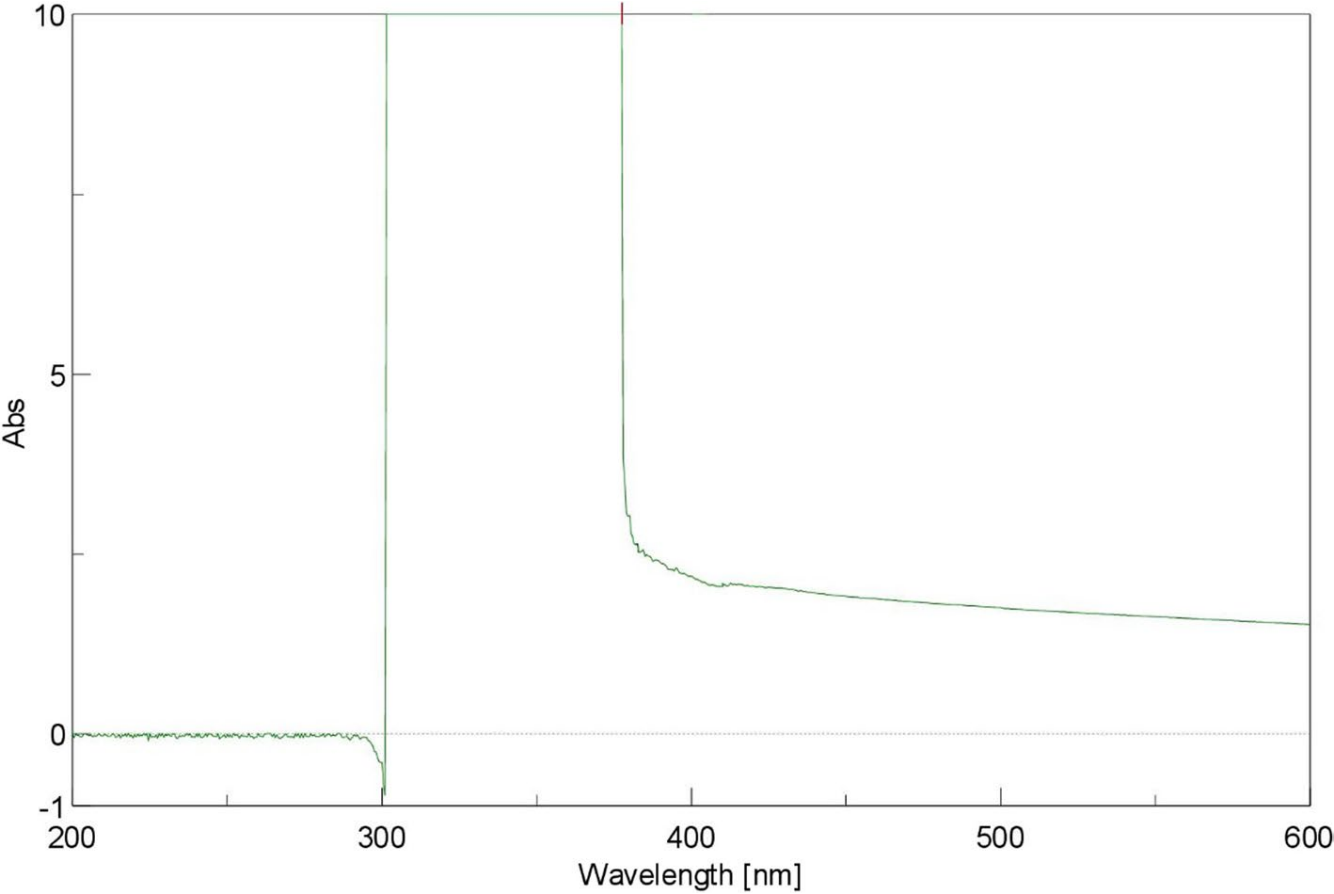
UV–visible spectroscopic analysis of synthesized ZnO NPs with a peak at 377 nm

**Fig. 2:**
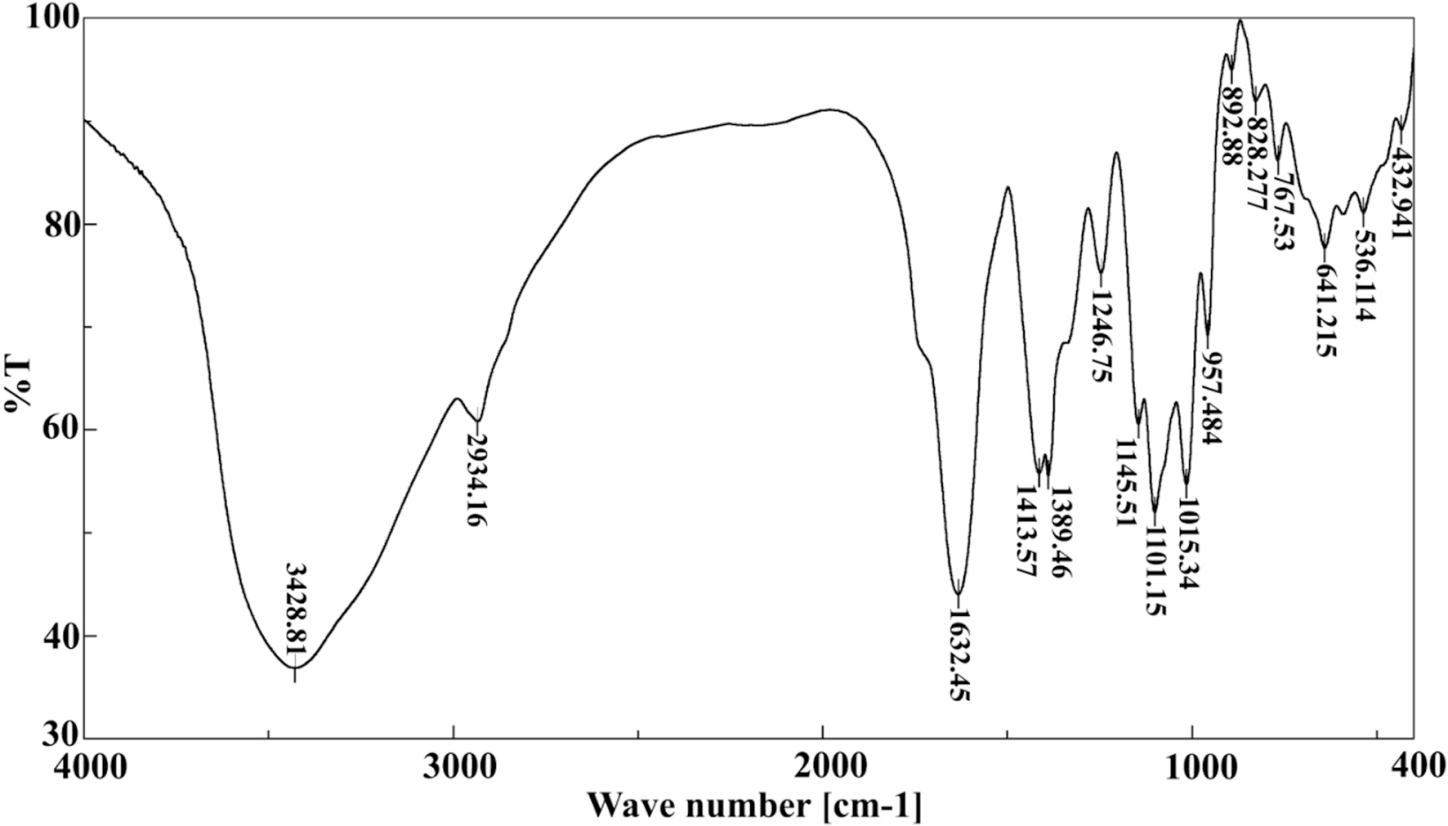
FTIR spectrum of biosynthesized ZnO NPs

**Fig. 3:**
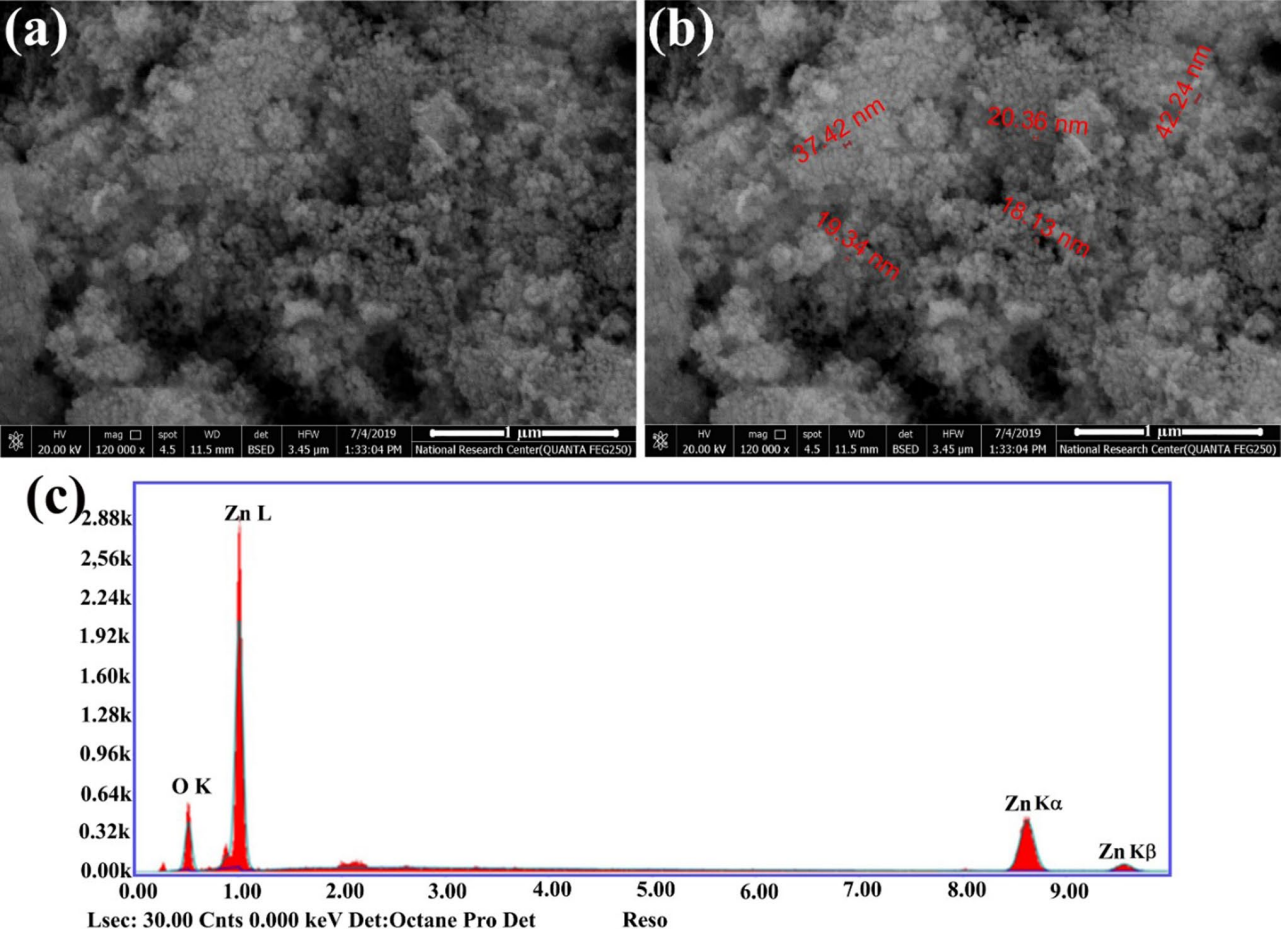
Scanning electron microscopic picture of ZnO NPs. SEM of ZnO NPs (**a**, **b**), SEM–EDS (**c**)

The UV–Vis spectra showed an absorption maximum peak at 377 nm that is a characteristic signature for ZnO NPs (Fig. 1). FTIR spectra showed intense peak at 3428.81, 2934.16, 1632.45, 1413.57, 1389.46, 1246.75, 1145.51, 1101.15, 1015.34, 957.48, 892.88, 828.27, 767.53, 641.21, 536.11, and 432.94 cm^−1^ for ZnO NPs (Fig. 2). The broad band at 3,428.81 cm^−1^ might be ascribed to the O–H group of water, phenol, and alcohol. The band at 2934.16 cm^−1^ corresponds to the C–H stretching of alkanes. The band observed at 1,632.45 cm^−1^ represents the C=C stretch of aromatic rings, as well as the C=O stretch of polyphenol compound. The band at 1413.57 cm^-1^ corresponds to the C–N stretching of the amide group. The band at 1389.46 cm^-1^ is attributed to the C=O bending of nitro groups. The vibrations of alkene C–H stretching caused the peak at 1246.7 cm^−1^. The 1015.34 cm^−1^ peak shows a stretch of aliphatic amines. The peak at 1101.15 cm^−1^ corresponds to the C–O bond that is returned to a specific group of alcohol and carboxylic acids. 892.88 cm^−1^ peak represents the C–C stretching of alkanes. The 828.27 and 767.53 cm^−1^ correspond to the C–N stretching of the amine group. The peak of zinc oxide nanostructures was appeared at 432.94–641.21 cm^−1^. The FTIR analysis evidences the presence of C=O, C=C, C–N, C–H, C–O, and O–H, group, that corresponds to the presence of polyphenols, carboxylic acid, polysaccharide, amino acid, and proteins that responsible for the reduction and stabilization of the synthesized nanoparticles. The SEM images of ZnO NPs showed sizes ranging between 18 and 42 nm with spherical-shaped nanoparticles (Fig. 3a, b). EDS analysis of ZnO NPs showed two picks: zinc 18.92% and oxygen 81.08% (Fig. 3c).

### Effect of ZnO NPs on the Embryonated Eggs

The ovicidal efficacy of ZnO NPs against the embryonated eggs is shown in Table 1. ZnO NPs showed significant mortality on the embryonated eggs comparing with the reference acaricide (Butox^®^5%) and control. The mortality percentage increased with the increased ZnO NPs concentration. In comparison with the reference acaricide Butox^®^5%, ZnO NPs showed higher ovicidal activity. At the highest concentration of 32 mg/ml, the mortality percentage was 88.2%. Moreover, the lowest concentration 4 mg/ml caused 11.3% mortality. The mortality percentage was 67.8 and 42.8%, at 16 and 8 mg/ml respectively. LC_50_ and LC_90_ were 11.69 and 32.64 mg/ml, respectively, as shown in Table 1.

**Table 1:** Acaricidal efficacy of zinc oxide nanoparticles against embryonated eggs, larvae, engorged nymphs, and unfed adults of *Hyalomma dromedarii*

Tick stage	Treatment	Conc	Mortality %	LC_50_ (mg/ml)	LC_90_ (mg/ml)	Slope ± SE
Embryonated eggs *F*_5,12_ = 77.696 *P* < 0.001	ZnO NPs (mg/ml)	32	88.2 ± 6.1^d^	11.69	32.64	2.8 ± 0.24
		16	67.8 ± 4.9^c^			
		8	42.8 ± 1.4^b^			
		4	11.3 ± 1.7^a^			
	Butox^®^5.0 (1 ml/l)	1	71.6 ± 4.4^ cd^			
	Control		6.6 ± 0.51^a^			
Larvae *F*_5,12_ = 79.035 *P* < 0.001	ZnO NPs (mg/ml)	32	100 ± 0.00^e^	8.03	34.41	2.02 ± 0.24
		16	82.6 ± 6.20^d^			
		8	45.7 ± 2.16^c^			
		4	29.1 ± 6.79^b^			
	Butox^®^5.0 (ml/l)	1	100 ± 0.00^e^			
	Control		0.00 ± 0.00^a^			
Engorged nymphs *F*_5,12_ = 29.689 *P* < 0.001	ZnO NPs (mg/ml)	16	85.00 ± 5.00^d^	3.96	17.35	2.00 ± 0.21
		8	75.0 ± 5.00^ cd^			
		4	56.6 ± 8.81^c^			
		2	23.3 ± 6.66^b^			
	Butox^®^5.0 (1 ml/l)	1	66.66 ± 6.66^ cd^			
	Control		0.00 ± 0.00^a^			
Unfed adult	ZnO NPs (mg/ml)	32	0.00	–	–	–
	Butox^®^5.0 (1 ml/l)	1	100			
	Control		0.00			

*LC*_*50*_ lethal concentration for 50% of individuals, *LC*_*90*_ lethal concentration for 90% of individuals, *Conc* Concentrations

^a,b,…,ect^The significant difference between the means of mortality percentages according to Duncan’s test (*P* < 0.05)

### Effect of ZnO NPs on Larvae

*Hyalomma dromedarii* larvae were sensitive to synthesized ZnO NPs 24 h after treatment (Table 1). The concentration of 32 mg/ml recorded 100% mortality. This result was the same as reference acaricide (Butox^®^5%). The concentrations of 16, 8, and 4 mg/ml resulted in a mortality percentage of 82.6, 54.7, and 29.1%, respectively. The calculated LC_50_ and LC_90_ of ZnO NPs were 8.03 and 34.41 mg/ml, respectively, as shown in Table 1.

### Effect of ZnO NPs on Engorged Nymphs

The obtained results showed that the molting of the engorged nymphs of *H. dromedarii* was greatly affected after treatment with ZnO NPs compared to the reference acaricide (Butox^®^ 5%) and control, as shown in Table 1. ZnO NPs showed higher mortality compared to Butox^®^ 5% especially in concentrations higher than 4 mg/ml. ZnO NPs showed 85% mortality at concentration of 16 mg/ml. LC_50_ and LC_90_ for ZnO NPs were 3.96 and 17.35 mg/ml, respectively (Table 1).

### Effect of ZnO NPs on Unfed Adults

*Hyalomma dromedarii* unfed adult did not record mortality to the concentration of 32 mg/ml of ZnO NPs. This result was similar to that in the control, while Butox^®^ 5% recorded 100% mortality of unfed adults (Table 1).

### Effect of ZnO NPs on the Engorged Females

Engorged females treated with ZnO NPs with the concentrations range 32 mg/ml to 4 mg/ml gave EPI range 0.485 ± 0.022 to 0.520 ± 0.022 comparing with 0.15 ± 0.017 for Butox^®^ 5.0 and 0.600 ± 0.054 for the control. Engorged females treated with ZnO NPs exhibited egg number ranged 4256.04 ± 179.96 to 4259.1 ± 459.96 comparing with 623.7 ± 226.3 eggs for Butox^®^ 5.0 and 5380.26 ± 96.68 eggs for the control (Table 2). The laid eggs from engorged females treated with ZnO NPs showed a lower hatchability percentage (48.5–89.5%) than the control (98.2%) as shown in Table 2. Statistical analyses revealed that the EPI of females treated with ZnO NPs recorded EPI insignificantly (*P* > 0.05) lower than that of control and significantly (*P* < 0.05) higher than Butox^®^ 5.0. The egg number laid by females exposed to ZnO NPs was significantly lower than control and significantly higher than Butox^®^ 5.0. Both ZnO NPs and Butox^®^ 5.0 significantly reduced (*P* < 0.05) hatchability percentage in comparison to that of control.

**Table 2:** Reproductive performance of *Hyalomma dromedarii* engorged females treated with zinc oxide nanoparticles

Item	Concentration	Reproductive performance		
		EPI	Egg number	Hatchability (%)
ZnO NPs (mg/ml)	32	0.485 ± 0.022^b^	4256.04 ± 179.96^bc^	48.53 ± 7.75^a^
	16	0.527 ± 0.025^b^	3252.8 ± 181.03^b^	66.67 ± 5.80^ab^
	8	0.530 ± 0.022^b^	3898.3 ± 380.84^b^	82.50 ± 3.65^bc^
	4	0.520 ± 0.022^b^	4259.1 ± 459.96^bc^	89.5 ± 3.29^bc^
Butox^®^5.0 (1 ml/l)	1	0.152 ± 0.017^a^	623.7 ± 226.3^a^	65.9 ± 12.4^ab^
Control		0.600 ± 0.054^b^	5380.26 ± 96.68^c^	98.27 ± 0.323^c^
F _5,48_		28.855	35.614	7.071
P		< 0.001	< 0.001	< 0.001

*EPI* egg productive index

^a,b,… etc^The significant difference between the mean values of each parameter according to Duncan’s test (*P* < 0.05)

### Toxic Effect of ZnO NPs on Swiss Albino Mice

#### Hematological Changes

Table 3 shows the hematological changes in the mice after oral administration of ZnO NPs suspension. The level of the WBCs 8.9 10^9^/l was higher in treated mice vs the control group, however, this difference was not statistically significant (*P* > 0.05), while the level of the RBCs 7.71 10^12^/l, HCT 39.9%, and platelet count 1245 10^9^/l were very similar between the groups. The solvent control showed similar values to those of the control.

**Table 3:** Hematological data of the mice administered five times oral dose of 844 mg/kg zinc oxide nanoparticles for 5 consecutive days

Group	WBC (10^9^/l)	RBC (10^12^/l)	HCT (%)	MCH (pg)	MCHC (g/dL)	PLT (10^9^/l)	Hb (g/dL)
ZnO NPs	8.90 ± 1.20	7.71 ± 0.50	39.93 ± 3.65	16.66 ± 0.31	32.33 ± 0.48	1245.00 ± 114.76	12.86 ± 1.0
Control	5.13 ± 1.68	7.45 ± 0.40	36.46 ± 1.32	16.03 ± 0.31	32.80 ± 0.41	1112.0 ± 197.4	11.93 ± 0.48^a^
Solvent control	5.90 ± 2.10	7.94 ± 0.37	40.56 ± 2.01	16.86 ± 0.08	33.03 ± 0.12	1125.66 ± 66.49	13.40 ± 0.70^ab^
*F*_2,6_	1.360	0.319	0.662	2.704	0.419	0.284	0.956
*P*	NS	NS	NS	NS	NS	NS	NS

*NS* Non-significant (*P* > 0.05)

#### Biochemical Changes

Table 4 shows the effects of the orally administered ZnO NPs on the liver enzymes Alkaline phosphatase (ALP) and Alanine aminotransferase (ALT); and their effects on creatinine. ALP (94.3 U/l) and ALT (86.6 U/l) levels were higher than those in the negative control group, although the difference was not statistically significant (*P* > 0.05). Furthermore, in the solvent control group, the levels of the ALP and ALT were 74 U/l and 41.6 U/l, respectively. For the renal parameter, the recorded creatinine level was similar in both ZnO NPs and solvent control 0.40 mg/dl which was non-significantly (*P* > 0.05) higher than that in the control group 0.33 mg/dl.

**Table 4:** Biochemical analysis of serum collected from mice administered five times oral dose of 844 mg/kg zinc oxide nanoparticles for 5 consecutive days

Group	ALP (U/l)	ALT (U/l)	Creatinine (mg/dl)
ZnO NPs	94.3 ± 17.3	86.6 ± 14.74	0.40 ± 0.00
Control	85 ± 5.7	67.0 ± 9.60	0.33 ± 0.033
Solvent control	74.0 ± 13.0	41.6 ± 7.17	0.40 ± 0.00
F_2,6_	1.644	4.226	4.000
P	NS	NS	NS

*ALP* alkaline phosphatase, *ALT* alanine aminotransferase

*NS* Non-significant (*P* > 0.05)

### Histopathological Changes of the Liver and Kidney of the Mice Treated with ZnO NPs

**Fig. 4:**
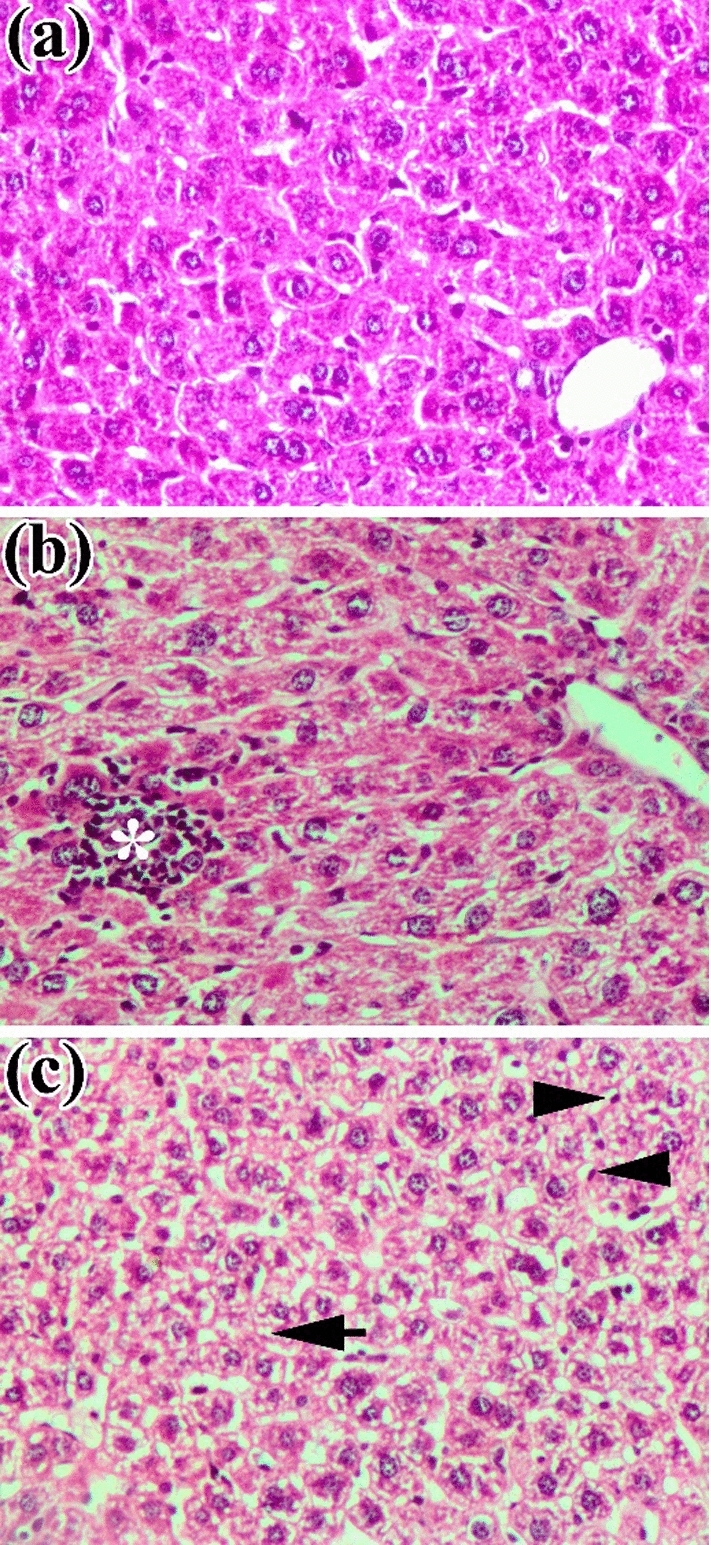
Histopathological changes in the liver of mice treated with ZnO NPs. **a** Control group showing the normal architecture of the liver. **b** Solvent control group showing aggregation of lymphocytes (white star). **c** ZnO NPs treated group showing cloudy swelling and vacuolar degeneration (black arrow) with activation of Kupffer cells (black head arrow). These micrographs were captured at a magnification of 200X

**Fig. 5:**
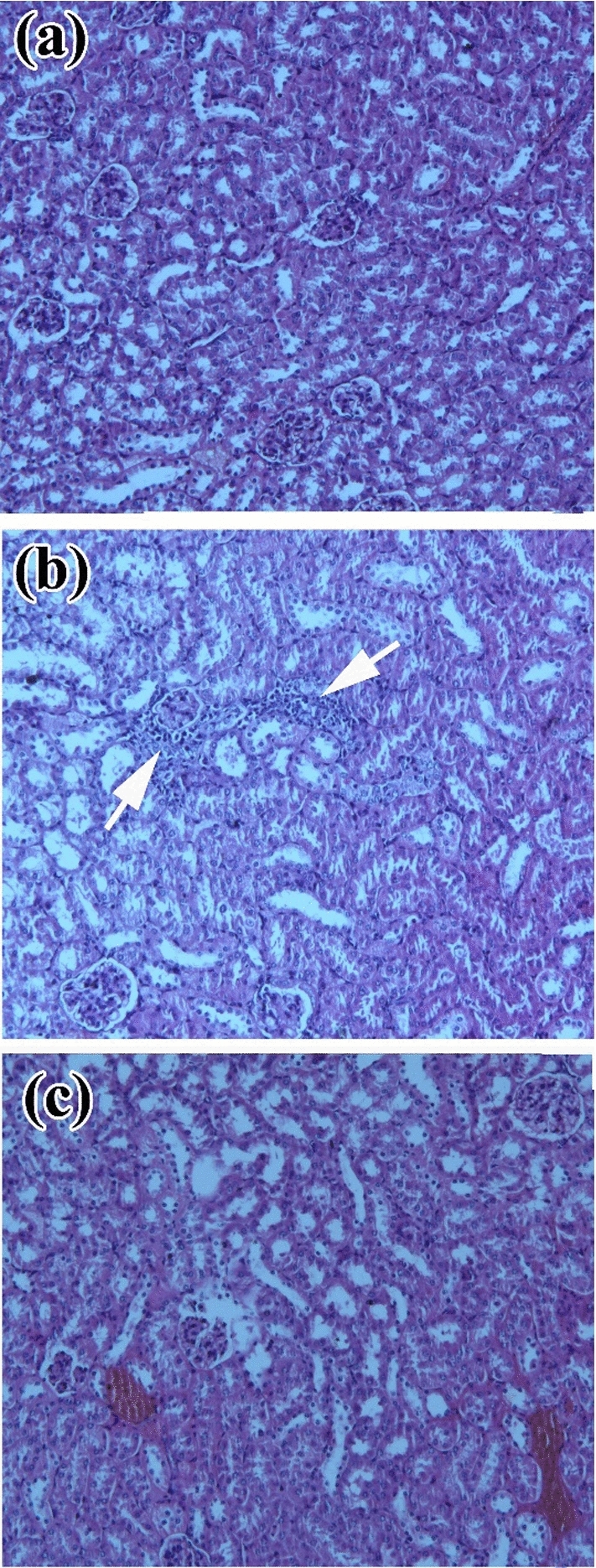
Histopathological changes in the kidney of the mice treated with ZnO NPs. **a** Control group showing normal features of the kidney. **b** Solvent control group showing aggregation of lymphocytes (white arrow). **c** ZnO NPs treated group showing hemolysis and degeneration of epithelial cells of tubules. These micrographs were captured at the magnification of 100×

The histopathological changes of the liver and kidney of the mice after oral uptake of ZnO NPs are shown in Figs. 4 and 5. The liver of the mice treated with ZnO NPs showed hepatic cells with mild degenerative changes mainly cloudy swelling and vacuolar degeneration with activation of Kupffer cells (Fig. 4c). The liver of the solvent control group showed mainly focal aggregation of lymphocytes in the hepatic parenchyma (white arrow) (Fig. 4b). The kidney of the ZnO NPs treated group showed degeneration of epithelial cells of tubules in addition to degenerated epithelium multiple areas of hemolysis appeared (Fig. 5c). Kidney tissue of the solvent control group showed mainly focal aggregation of lymphocytes (white arrow) (Fig. 5b).

## Discussion

As a result of the problems associated with the indiscriminative use of the chemical acaricides, like the development of resistance and environmental pollution [36], there is a need for a novel strategy concerning tick control to restrict the problems of chemical acaricides. Nanoparticles, including metal oxide NPs, are considered a novel pesticide against arthropod pests [37, 38]. Therefore, in the current study, the acaricidal activity of synthesized ZnO NPs as an alternative for *H. dromedarii* control was evaluated. Several studies on the effect of metal and metal oxide NPs on different species of ticks have been performed. Most of the previous studies evaluated the effect of synthesized NPs against only the larval stage of ticks as the study of Marimuthu *et al.* [39] evaluated the effect of green-synthesized titanium dioxide (TiO_2_) NPs on *Rhipicephalus (Boophilus) microplus* larvae with LC_50_ value of 5.43 mg/l. Another one conducted by Rajakumar *et al.* [40] evaluated the effect of green-synthesized TiO_2_ NPs against *Hyalomma anatolicum* larvae with an LC_50_ value of 4.11 mg/l. In addition to Rajakumar and Rahuman [41] used *R. (B) microplus* larvae to evaluate the efficacy of Ag NPs produced by an aqueous extract of *Manilkara zapota* with LC_50_ of 3.44 mg/l. In comparison with the results of the present study, the toxicity of nanomaterials tested against larvae of both *R. microplus* and *H. anatolicum* was higher. This may attribute to the nature of these materials or the larvae of the tick species that may be more susceptible to NPs than *H. dromedarii* larvae.

The current study evaluated the acaricidal efficacy of the green-synthesized ZnO NPs using *M. azedarach* ripened fruit extract against different developmental stages of *H. dromedarii*. Only a few studies evaluated the effect of the green-synthesized ZnO NPs on species of ticks other than *H. dromedarii.* Gandhi *et al.* [42] reported the acaricidal effect of ZnO NPs produced by the green synthesization method against *R. microplus* larvae and the LC_50_ was 6.87 mg/l. Another study evaluated the effect of green-synthesized ZnO NPs against *R. microplus* larvae with an LC_50_ of 0.0017 mg/ml [20]. In contrary with our finding, the toxicity of ZnO NPs against larvae recorded by these authors might attribute to the method used in preparing the nanomaterials that make it more toxic than that used in this study as well as *R. microplus* larvae may be more sensitive than *H. dromedarii* larvae. As previously mentioned by Abdel-Ghany *et al.* [29], prospective tick control should include all stages in the life cycle of the ticks (on and off the host). Therefore, this study evaluated the effect of ZnO NPs on the active stages (on the host); larvae, unfed adults, engorged females, and dormant stages; embryonated eggs and engorged nymphs of *H. dromedarii*. In comparing with the reference acaricide (Butox^®^5.0), ZnO NPs showed higher activity against embryonated eggs and engorged nymphs at the concentrations of 32 mg/ml and 16 mg/ml resulting in 88.2% and 85% mortality, respectively. This may attribute to higher penetration of NPs throughout the surfaces of dormant stages such as eggs and engorged nymphs than active stage as adults. Furthermore, both Butox^®^5.0 and ZnO NPs (32 mg/ml) recorded 100% mortality against larvae. The calculated LC_50_ value for embryonated eggs, larvae, and engorged nymphs were 11.6 mg/ml, 8.03 mg/ml, and 3.9 mg/ml, respectively, which confirmed that the synthesized ZnO NPs have a potent effect on engorged nymphs followed by larvae then eggs. The high sensitivity of larvae to NPs may be due to its small size and soften alloscutum that allow NPs to penetrate rapidly and killed it.

In this study, the acaricidal activity of ZnO NPs on the larvae agreed with the study of Kirthi *et al.* [19] who found that the acaricidal activity of ZnO NPs synthesized by wet chemical route against the larvae of *R. (B.) microplus* recorded 100% mortality at concentrations of 10 mg/l. Ramyadvi *et al.* [43] evaluated the efficacy of copper NPs synthesized by polyol process against *R. microplus* larvae with LC_50_ (1.06 mg/l). Ni NPs synthesized by chemical route showed antiparasitic activity against *H. anatolicum* and *R.* (*B*.) *microplus* larvae with 100% mortality at 25 mg/l [44]. For unfed adults, ZnO NPs did not record mortality even at the highest concentration of 32 mg/ml; this may return to the highly chitinized cuticles of the unfed adults while Butox®5.0 recorded 100% mortality for unfed adults. These results were similar to the results of Abdel-Ghany *et al*. [34], where NiO NPs did not record mortality for unfed adults at the concentration of 32 mg/ml. In this study, we observed that Butox^®^5.0 has a low effect on dormant stages as embryonated eggs and engorged nymphs than active stages as larvae, unfed adults, and full-engorged females. In the author’s opinion, this may return to the mode of action of Butox^®^5.0 which acts on the nervous system. It disrupts the nerve signals that might be more in the active stages than in the dormant stages.

Concerning the full-engorged females, ZnO NPs did not cause mortality for engorged females that succeeded to life after treatment and laid normal egg mass and egg number slightly lower than that in control, but a significant effect was observed in hatchability. At the highest concentration of 32 mg/ml, the EPI 0.485, *P* > 0.05, egg number 4256.04, *P* > 0.05, and hatchability 48.53%, *P* < 0.05 comparing with the control group, EPI 0.600, egg number 5380.26, and hatchability 98.27%. The effect of Butox^®^5.0 was in opposition to the effect of ZnO NPs where Butox^®^5.0 has a high effect on EPI and egg number and equal effect with 16 mg/ml on hatchability those recorded around 66%. These results were contrary with the results of Abdel-Ghany *et al*. [34]*.* who evaluated the effect of NiO NPs on full-engorged females of *H. dromedarii* where NiO NPs at a concentration of 32 mg/ml significantly affect EPI 0.377, *P* < 0.05, egg number 3627.4, *P* < 0.05, and hatchability 58.86, *P* < 0.05. Another study conducted by Arafa *et al.* [45] evaluated the antiparasitic efficacy of Ag NPs and ZnO NPs produced by a hydrothermal and chemical method on the *R. annulatus* adult ticks. They found that Ag NPs (400 mg/l) and ZnO NPs (8 g/l) showed 20% mortality of the engorged females in addition to a reduction of the egg number by 20.34%. Furthermore, Norouzi *et al.* [46] evaluated the acaricidal effect of Zn NPs synthesized by evaporation process against *Hyalomma* spp. at a concentration of 250 mg/ml which recorded 87.7% mortality by contact method and 100% mortality by spraying methods. A previous investigation by Norouzi *et al.* [47] evaluated the acaricidal efficacy of iron oxide against *Hyalomma* spp adults comparing two methods of exposure, spraying and contact methods. The mortality percentages were 85.7% and 71.5% for spraying and contact method, respectively, at the concentration of 250 μg/ml.

UV–Vis spectroscopic analysis showed a maximum absorption peak at 377 nm. This result was in agreement with the result obtained by Jayappa *et al.* [48] who utilized stem aqueous extract of *Mussaenda frondosa* for the green synthesis of ZnO NPs. This result indicated that secondary metabolites of *M. azedarach* fruits have a role in the phytosynthesis of ZnO NPs.

FTIR analysis of synthesized ZnO NPs showed a band at 2,934.16 cm^−1^ corresponding to the C–H stretching of alkanes. The 892.88 cm^−1^ peak represents the C–C stretching of alkanes. The band at 1413.57 cm^−1^ corresponds to the C–N stretching of the amide group [20]. The band observed at 1632.45 cm^−1^ represents the C=C stretch in the aromatic ring, and C=O stretch in polyphenols compounds [49]. The band at 1,389.46 is attributed to the C=O bending of nitro groups [50]. The peak of zinc oxide nanostructures was appeared at 432.94–641.21 cm^−1^ [51]. Thus, from the FTIR analyses, the aqueous extract of *M. azedarach* has a higher quantity of polyphenols, carboxylic acid, polysaccharide, amino acid, and protein which help in the stabilization of the synthesized NPs. ZnO NPs appeared spherical in shape with sizes ranging between 18 and 42 nm that determined by SEM. EDS analysis revealed that the sample has only zinc and oxygen. These obtained results were in accordance with Anbuvannan *et al.* [52] who found ZnO NPs with spherical shape and size range 20–40 nm after green synthesis using leaf extract of *Anisochilus carnosus.*

As ZnO NPs are increasingly used in different commercial products, it is necessary to study their toxicological effects. In the current study, an *in vivo* experiment was conducted on mice to investigate the ability of ZnO NPs to induce hematological and biochemical changes. Additionally, the histopathological examination may detect the abnormalities of the liver and kidney after uptake of ZnO NPs by the gastrointestinal tract. Although ZnO NPs will be applied topically to the animals, it was preferred to study the harmful effect orally to be sure about the effect of ZnO NPs safe or not if the animal makes licking of these materials during application. As the absorption is higher through the gut than the skin, if it appeared safe when administered orally, it will approximately be safe when applied topically.

The hematological parameters showed a higher level of WBCs in the mice exposed to ZnO NPs than untreated mice (control) although the differences were not statistically significant, while the level of RBCs, HCT, Hb, and platelet was nearly similar to the control group. These results were consistent with a work done by Choi *et al.* [53] who administered rats 30 mg/kg of ZnO NPs as a single dose which revealed an increase in the level of WBCs and lymphocytes with a normal level of RBCs, HCT, Hb, and platelet count. Another study conducted by Ben-Slama *et al.* [54] evaluated the sub-acute oral toxicity of ZnO NPs on rats after repeated oral administration of 10 mg/kg for 5 consecutive days resulting in normal RBC, WBC, hematocrit, Hb, and platelet count.

The level of ALT, AST, and ALP in the serum can be sensitive indicators for damage or diseased liver. Therefore, increased levels of these enzymes in mice treated with NPs indicated that the NPs caused hepatic damage [55]. In this study, the biochemical analysis of the serum of mice treated with ZnO NPs indicated an increase in the level of ALP, ALT, and creatinine although statistically non-significant *P* > 0.05. The histopathological examination of the liver and kidney showed signs of cytotoxicity as inflammatory response, vacuolar degeneration, and activation of Kupffer cells in the liver and the kidney suffered degeneration of epithelial cells of tubules in addition to multiple areas of hemolysis appeared. These findings were similar to an investigation of Esmaeillou *et al.* [56] who evaluated the acute oral toxicity of ZnO NPs (333.3 mg*/*kg) for 5 successive days in mice, revealed an increased level of ALT and AST, cellular necrosis, and congestion of the liver as well as hydropic degeneration and necrosis of the epithelial cells of the kidney tubules. Furthermore, a previous study conducted by Wang *et al.* [57] found an increase in the level of ALP and ALT in serum, edema, and degeneration of hepatocyte in the marginal area of the liver and around the central vein, in addition to the appearance of a spot of proteinaceous casts in the renal tubule after oral uptake of 20 nm and 120 nm ZnO NPs for 14 days. When the mice were treated with ZnO NPs (300 mg/kg) for 14 days, ALT and ALP levels increased in serum and cellular injury appeared in the liver [58]. After oral uptake of ZnO NPs for 14 days, impairment of mitochondria and cell membranes in the kidneys of the rat occurred [59]. ZnO NPs toxicity may significantly differ according to the type of the particle. The results from the previous studies suggested that the bioavailability of ZnO NPs is low after oral administration. The rats were orally dosed with 2000 mg/kg of ZnO NPs, C_max_ was only 179.5 μg/ml at the highest dose. However, most of the ZnO NPs administered might not be absorbed but excreted via feces, so the toxic effect after oral uptake was low [60]. In the author’s view, the future application of ZnO NPs used in this study for controlling the camel tick *H. dromedarii* will be through an external route by spraying on the external surface of the animal body or dipping the animal in a bath. This consequently allows little quantities of applied NPs to be absorbed, so their toxicity will be lower than other routes of administration; this should be further evaluated. In addition, the mice results should be validated and assessed in camels.

## Conclusion

The acaricidal activity of synthesized ZnO NPs against different developmental stages of the camel ticks *H. dromedarii* was evaluated. ZnO NPs have good efficacy against eggs, larvae, and engorged nymphs. ZnO NPs minimized the number of eggs laid by engorged females and the hatchability of their eggs. ZnO NPs did not affect unfed adults. Furthermore, the toxicity results of the mice revealed insignificant changes in the hemogram, biochemistry, with liver and kidney suffering few histopathological changes. Based on these findings, ZnO NPs may be incorporated in the control of camel tick *H. dromedarii.*

## References

[1] Yin H, Chen R, Casey PS, Ke PC, Davis TP, Chen C (2015). Reducing the cytotoxicity of ZnO nanoparticles by a pre-formed protein corona in a supplemented cell culture medium. RSC Adv.

[2] Prasad AR, Garvasis J, Oruvil SK, Joseph A (2019). Bio-inspired green synthesis of zinc oxide nanoparticles using *Abelmoschus esculentus mucilage* and selective degradation of cationic dye pollutants. J Phys Chem Solids.

[3] Goswami M, Bhattacharjee ANC (2018). Effect of annealing temperatures on the structural and optical properties of zinc oxide nanoparticles prepared by chemical precipitation method. Optik.

[4] Ishwarya R, Vaseeharan B, Kalyani S, Banumathi B, Govindarajan M, Alharbi NS, Benelli G (2018). Facile green synthesis of zinc oxide nanoparticles using *Ulva lactuca* seaweed extract and evaluation of their photocatalytic, antibiofilm and insecticidal activity. J Photochem Photobiol B.

[5] Saravanan M, Gopinath V, Chaurasia MK, Syed A, Ameen F, Purushothaman N (2018). Green synthesis of anisotropic zinc oxide nanoparticles with antibacterial and cyto-friendly properties. Microb Pathog.

[6] Santhoshkumar T, Rahuman AA, Bagavan A, Marimuthu S, Jayaseelan C, Kirthi AV, Velayutham K (2012). Evaluation of stem aqueous extract and synthesized silver nanoparticles using *Cissus quadrangularis* against *Hippobosca maculata* and *Rhipicephalus (Boophilus) microplus*. Exp Parasitol.

[7] Shaikh F, Panhwar QK, Balouch A, Ali S, Panhwar WA, Sheikh F (2020). Synthesis of zinc oxide nanoparticles and their functionalization with chrysin: exploration of its applications. Int J Environ Anal Chem.

[8] Pugazhendhi A, Prabhu R, Muruganantham K, Shanmuganathan R, Natarajan S (2019). Anticancer, antimicrobial and photocatalytic activities of green synthesized magnesium oxide nanoparticles (MgO NPs) using aqueous extract of *Sargassum wightii*. J Photochem Photobiol B Biol.

[9] Rubae AAY (2009). The potential uses of *Melia azedarach* L. as pesticidal and medicinal plant, review. Am Eurasian J Sustain Agric.

[10] Manokari M, Ravindran CP, Shekhawat MS (2016). Biosynthesis of zinc oxide nanoparticles using *Melia azedarach* L. extracts and their characterization. Int J Pharm Sci Res.

[11] Mehmood A, Murtaza G, Bhatti TM, Kausar R (2017). Phyto-mediated synthesis of silver nanoparticles from *Melia azedarach* L. leaf extract: characterization and antibacterial activity. Arab J Chem.

[12] Khan TM, Mateen A (2018). Synthesis of CuO nanoparticles by using leaf extracts of *Melia azedarach* and *Morus nigra* and their antibacterial activity. J Innov Sci.

[13] Elango G, Rahuman AA (2011). Evaluation of medicinal plant extracts against ticks and fluke. Parasitol Res.

[14] Tian ZC, Liu GY, Xie JR, Yin H, Luo JX, Luo ZLY, J,  (2011). Discrimination between *Haemaphysalis longicornis* and *H. qinghaiensis* based on the partial 16S rDNA and the second internal transcribed spacer (ITS-2). Exp Appl Acarol.

[15] Zahir AA, Rahuman AA, Bagavan A, Santhoshkumar T, Mohamed RR, Kamaraj C, Marimuthu S (2010). Evaluation of botanical extracts against *Haemaphysalis bispinosa Neumann* and *Hippobosca maculata* Leach. Parasitol Res.

[16] Abdel-Shafy S, Soliman MM, Habeeb SM (2007). In vitro acaricidal effect of some crude extracts and essential oils of wild plants against certain tick species. Acarologia.

[17] Pirali-Kheirabadi KH, da Silva JT (2011). In vitro assessment of the acaricidal properties of *Artemisia annua* and *Zataria multiflora* essential oils to control cattle ticks. Iran J Parasitol.

[18] Underwood C, Van Eps AW (2012). Nanomedicine and veterinary science: the reality and the practicality. Vet J.

[19] Kirthi AV, Rahuman AA, Rajakumar G, Marimuthu S, Santhoshkumar T, Jayaseelan C, Velayutham K (2011). Acaricidal, pediculocidal and larvicidal activity of synthesized ZnO nanoparticles using wet chemical route against blood feeding parasites. Parasitol Res.

[20] Banumathi B, Malaikozhundan B, Vaseeharan B (2016). In vitro acaricidal activity of ethnoveterinary plants and green synthesis of zinc oxide nanoparticles against *Rhipicephalus (Boophilus) microplus*. Vet Parasitol.

[21] Kancharana S, Chengalva RV, Kothapalli SR, Yegireddy M, Bollini S, Vara PTNVK (2020). Assessment of acaricidal activity of nanoscale ZnO encapsulated piperine formulation against *Rhipicephalus microplus*. IET Nanobiotechnol.

[22] Koeneman BA, Zhang Y, Westerhoff P, Chen Y, Crittenden JC, Capco DG (2010). Toxicity and cellular responses of intestinal cells exposed to titanium dioxide. Cell Biol Toxicol.

[23] Johnston HJ, Hutchison G, Christensen FM, Peters S, Hankin S, Stone V (2010). A review of the in vivo and in vitro toxicity of silver and gold particulates: particle attributes and biological mechanisms responsible for the observed toxicity. Crit Rev Toxicol.

[24] Cui Y, Liu H, Zhou M, Duan Y, Li N, Gong X, Hong F (2011). Signaling pathway of inflammatory responses in the mouse liver caused by TiO_2_ nanoparticles. J Biomed Mater Res Part A.

[25] El-Hadidi MN, Boulos L (1988) The street trees of Egypt (No. Revised Ed.). American University in Cairo Press

[26] Umar H, Kavaz D, Rizaner N (2019). Biosynthesis of zinc oxide nanoparticles using *Albizia lebbeck* stem bark, and evaluation of its antimicrobial, antioxidant, and cytotoxic activities on human breast cancer cell lines. Int J Nanomed.

[27] Walker AR, Bouattour A, Camicas JL, Estrada-Pena A, Horak IG, Latif AA, Pegram RG, Preston PM (2003). Ticks of domestic animals in Africa: a guide to identification of species. Biosci Rep Edinburgh.

[28] Abdel-Ghany HS, Abdel-Shafy S, Abuowarda M, El-Khateeb RM, HoballahEM FMM (2021). Acaricidal activity of *Artemisia herba-alba* and *Melia azedarach* oil nano-emulsion against *Hyalomma dromedarii* and their toxicity on Swiss albino mice. Exp Appl Acarol.

[29] Abdel-Ghany HS, Fahmy MM, Abuowarda MM, Abdel-Shafy S, El-Khateeb RM, Hoballah EM (2019). In vitro acaricidal effect of *Melia azedarach* and *Artemisia herba-alba* extracts on *Hyalomma dromedarii* (Acari: Ixodidae): embryonated eggs and engorged nymphs. J Parasitic Dis.

[30] Abdel-Ghany HSM, Abdel-Shafy S, Abuowarda MM, El-Khateeb RM, Hoballah EM, Fahmy MM (2021). Acaricidal activity of some medicinal plant extracts against different developmental stages of the camel tick *Hyalomma dromedarii*. Adv Anim Vet Sci.

[31] Drummond RO, Ernst SE, Trevino JL, Gladney WJ, Graham OH (1973). *Boophilus annulatus* and *B. microplus*: laboratory tests of insecticides. J Econ Entomol.

[32] Abuowarda MM, Haleem MA, Elsayed M, Farag H, Magdy S (2020). Bio-pesticide control of the brown dog tick (*Rhipicephalus sanguineus*) in Egypt by using two entomopathogenic fungi (*Beauveria bassiana* and *Metarhizium anisopliae*). Int J Vet Sci.

[33] Mesallam DI, Deraz RH, Aal SMA, Ahmed SM (2019). Toxicity of subacute oral zinc oxide nanoparticles on testes and prostate of adult albino rats and role of recovery. J Histol Histopathol.

[34] Abdel-Ghany HS, Abdel-Shafy S, Abuowarda MM, El-Khateeb RM, Hoballah E, Hammam AMM, Fahmy MM (2021). In vitro acaricidal activity of green synthesized nickel oxide nanoparticles against the camel tick, *Hyalomma dromedarii* (Ixodidae), and its toxicity on Swiss albino mice. Exp Appl Acarol.

[35] Finney DJ (1962). Probit analysis a statistical treatment of the response curve.

[36] Ghosh S, Tiwari SS, Srivastava S, Sharma AK, Kumar S, Ray DD, Rawat AK (2013). Acaricidal properties of *Ricinus communis* leaf extracts against organophosphate and pyrethroids resistant *Rhipicephalus* (*Boophilus*) *microplus*. Vet Parasitol.

[37] Santhoshkumar T, Rahuman AA, Rajakumar G, Marimuthu S, Bagavan A, Kamaraj JC (2011). Synthesis of silver nanoparticles using *Nelumbo nucifera* leaf extract and its larvicidal activity against malaria and filariasis vectors. Parasitol Res.

[38] Athanassiou CG, Kavallieratos NG, Benelli G, Losic D, Usha Rani P, Desneux N (2018). Nanoparticles for pest control: current status and future perspectives. J Pest Sci.

[39] Marimuthu S, Rahuman AA, Jayaseelan C, Kirthi AV, Santhoshkumar T, Velayutham K, Bagavan A, Kamaraj C, Elango G, Iyappan M, Siva C, Karthik L, Bhaskara Rao KV (2013). Acaricidal activity of synthesized titanium dioxide nanoparticles using *Calotropis gigantea* against *Rhipicephalus microplus* and *Haemaphysalis bispinosa*. Asian Pac J Trop Med.

[40] Rajakumar G, Rahuman AA, Jayaseelan C, Santhoshkumar T, Marimuthu S, Kamaraj C, Bagavan A, Zahir AA, Kirthi AV, Elango G, Arora P, Karthikeyan R, Manikandan S, Jose S (2014). *Solanum trilobatum* extract-mediated synthesis of titanium dioxide nanoparticles to control *Pediculus humanus capitis*, *Hyalomma anatolicum anatolicum* and *Anopheles subpictus*. Parasitol Res.

[41] Rajakumar G, Rahuman AA (2012). Acaricidal activity of aqueous extract and synthesized silver nanoparticles from *Manilkara zapota* against *Rhipicephalus (Boophilus) microplus*. Res Vet Sci.

[42] Gandhi PR, Jayaseelan C, Mary RR, Mathivanan D, Suseem SR (2017). Acaricidal, pediculicidal and larvicidal activity of synthesized ZnO nanoparticles using *Momordica charantia* leaf extract against blood-feeding parasites. Exp Parasitol.

[43] Ramyadevi J, Jeyasubramanian K, Marikani A, Rajakumar G, Rahuman AA, Santhoshkumar T, Kirthi AV, Jayaseelan C, Marimuthu S (2011). Copper nanoparticles synthesized by polyol process used to control hematophagous parasites. Parasitol Res.

[44] Rajakumar G, Rahuman AA, Velayutham K, Ramyadevi J, Jeyasubramanian K, Marikani A, Zahir AA (2013). Novel and simple approach using synthesized nickel nanoparticles to control blood-sucking parasites. Vet Parasitol.

[45] Arafa WM, Mohammed AN, El-Ela FIA (2019). Acaricidal efficacy of deltamethrin-zinc oxide nanocomposite on *Rhipicephalus (Boophilus) annulatus* tick. Vet Parasitol.

[46] Norouzi R, Ataei A, Hejazy M, Shahbazi P (2019). Acaricidal activity of zinc oxide nanoparticles against *Hyalomma* spp. in vitro. Nanomed Res J.

[47] Norouzi R, Kazemi F, Siyadatpanah A (2020). Acaricidal effect of Iron nanoparticles against *Hyalomma* spp. in vitro. J Zoon Dis.

[48] Jayappa MD, Ramaiah CK, Kumar MAP, Suresh D, Prabhu A, Devasya RP, Sheikh S (2020). Green synthesis of zinc oxide nanoparticles from the leaf, stem and in vitro grown callus of *Mussaenda frondosa* L. characterization and their applications. Appl Nanosci.

[49] Awwad AM, Albiss B, Ahmad AL (2014). Green synthesis, characterization and optical properties of zinc oxide nanosheets using *Olea europea* leaf extract. Adv Mater Lett.

[50] Senthilkumar SR, Sivakumar T (2014). Green tea (*Camellia sinensis*) mediated synthesis of zinc oxide (ZnO) nanoparticles and studies on their antimicrobial activities. Int J Pharm Pharm Sci.

[51] Sangeetha G, Rajeshwari S, Venckatesh R (2011). Green synthesis of zinc oxide nanoparticles by *aloe barbadensis miller* leaf extract: structure and optical properties. Mater Res Bull.

[52] Anbuvannan M, Ramesh M, Viruthagiri G, Shanmugam N, Kannadasan N (2015). *Anisochilus carnosus* leaf extract mediated synthesis of zinc oxide nanoparticles for antibacterial and photocatalytic activities. Mater Sci Semicond Process.

[53] Choi J, Kim H, Kim P, Jo E, Kim HM, Lee MY, Park K (2015). Toxicity of zinc oxide nanoparticles in rats treated by two different routes: single intravenous injection and single oral administration. J Toxicol Environ Health Part A.

[54] Ben-Slama I, Mrad I, Rihane N, Mir LE, Sakly M, Amara S (2015). Sub-acute oral toxicity of zinc oxide nanoparticles in male rats. J Nanomed Nanotechnol.

[55] Wang B, Feng WY, Wang TC, Jia G, Wang M, Shi JW, Zhang F, Zhao YL, Chai ZF (2006). Acute toxicity of nano and micro-scale zinc powder in healthy adult mice. Toxicol Lett.

[56] Esmaeillou M, Moharamnejad M, Hsankhani R, Tehrani AA, Maadi H (2013). Toxicity of ZnO nanoparticles in healthy adult mice. Environ Toxicol Pharmacol.

[57] Wang B, Feng W, Wang M, Wang T, Gu Y, Zhu M, Wang J (2008). Acute toxicological impact of nano-and sub-micro-scaled zinc oxide powder on healthy adult mice. J Nanopart Res.

[58] Sharma V, Singh P, Pandey AK, Dhawan A (2012). Induction of oxidative stress, DNA damage and apoptosis in mouse liver after sub-acute oral exposure to zinc oxide nanoparticles. Mutat Res.

[59] Yan G, Huang Y, Bu Q, Deng P, Zhou J, Zhao Y (2012). Zinc oxide nanoparticles cause nephrotoxicity and kidney metabolism alterations in rats. J Environ Sci Health Part A.

[60] Baek M, Chung HE, Yu J, Lee JA, Kim TH, Oh JM, Choi SJ (2012). Pharmacokinetics, tissue distribution, and excretion of zinc oxide nanoparticles. Int J Nanomed.

